# Early relapse is an adverse prognostic factor for survival outcomes in patients with oral cavity squamous cell carcinoma: results from a nationwide registry study

**DOI:** 10.1186/s12885-023-10602-1

**Published:** 2023-02-07

**Authors:** Chi-Ying Tsai, Yu-Wen Wen, Shu-Ru Lee, Shu-Hang Ng, Chung-Jan Kang, Li-Yu Lee, Chuen Hsueh, Chien-Yu Lin, Kang-Hsing Fan, Hung-Ming Wang, Chia-Hsun Hsieh, Chih-Hua Yeh, Chih-Hung Lin, Chung-Kan Tsao, Tuan-Jen Fang, Shiang-Fu Huang, Li-Ang Lee, Ku-Hao Fang, Yu-Chien Wang, Wan-Ni Lin, Li-Jen Hsin, Tzu-Chen Yen, Nai-Ming Cheng, Chun-Ta Liao

**Affiliations:** 1 Department of Oral and Maxillofacial Surgery, Chang Gung Memorial Hospital, Chang Gung University, Taoyuan, Taiwan, ROC; 2 Clinical Informatics and Medical Statistics Research Center, Chang Gung University, Taoyuan, Taiwan, ROC; 3 Research Service Center for Health Information, Chang Gung University, Taoyuan, Taiwan, ROC; 4 Department of Diagnostic Radiology, Chang Gung Memorial Hospital, Chang Gung University, Taoyuan, Taiwan, ROC; 5 Department of Otorhinolaryngology, Head and Neck Surgery, Chang Gung Memorial Hospital, Chang Gung University, Taoyuan, Taiwan, ROC; 6 Department of Pathology, Chang Gung Memorial Hospital, Chang Gung University, Taoyuan, Taiwan, ROC; 7 Department of Radiation Oncology, Chang Gung Memorial Hospital, Chang Gung University, Taoyuan, Taiwan, ROC; 8 Department of Medical Oncology, Chang Gung Memorial Hospital, Chang Gung University, Taoyuan, Taiwan, ROC; 9 Department of Plastic and Reconstructive Surgery, Chang Gung Memorial Hospital and Chang Gung University, Taoyuan, Taiwan, ROC; 10 Department of Nuclear Medicine and Molecular Imaging Center, Chang Gung Memorial Hospital, Chang Gung University, Taoyuan, Taiwan, ROC; 11 Division of Thoracic Surgery, Chang Gung Memorial Hospital, Taoyuan, Taiwan, ROC; 12 Department of Otorhinolaryngology, Head and Neck Surgery, Linkou Chang Gung Memorial Hospital, Chang Gung University, at Linkou, No. 5, Fu-Hsing ST., Kwei-Shan, Taoyuan, Taiwan

**Keywords:** Oral cavity squamous cell carcinoma, Relapse interval, Cancer registry, Propensity score matching, Survival outcomes

## Abstract

**Background:**

The prognostic significance of the relapse interval in patients with resected oral cavity squamous cell carcinoma (OCSCC) is a matter of ongoing debate. In this large-scale, registry-based, nationwide study, we examined whether the time interval between surgery and the first disease relapse may affect survival outcomes in Taiwanese patients with OCSCC.

**Methods:**

Data made available by the Taiwan Health Promotion Administration as of 2004 were obtained. The study cohort consisted of patients who were included in the registry between 2011 and 2017. Disease staging was performed according to the American Joint Committee on Cancer (AJCC) Staging Manual, Eight Edition. We retrospectively reviewed the clinical records of 13,789 patients with OCSCC who received surgical treatment. A total of 2327 (16.9%) patients experienced a first disease relapse. The optimal cutoff value for the relapse interval was 330 days when both 5-year disease-specific survival (DSS) and overall survival (OS) (≤ 330/>330 days, n = 1630/697) were taken into account. In addition, we undertook a propensity score (PS)-matched analysis of patients (n = 654 each) with early (≤ 330 days) *versus* late (> 330 days) relapse.

**Results:**

The median follow-up time in the entire study cohort was 702 days (433 and 2001 days in the early and late relapse groups, respectively). Compared with patients who experienced late relapse, those with early relapse showed a higher prevalence of the following adverse prognostic factors: pT4, pN3, pStage IV, poor differentiation, depth of invasion ≥ 10 mm, and extra-nodal extension. Multivariable analysis revealed that early relapse was an independent adverse prognostic factor for both 5-year DSS and OS (average hazard ratios [AHRs]: 3.24 and 3.91, respectively). In the PS-matched cohort, patients who experienced early relapse showed less favorable 5-year DSS: 58% *versus* 30%, *p* < 0.0001 (AHR: 3.10 [2.69 − 3.57]) and OS: 49% *versus* 22%, *p* < 0.0001 (AHR: 3.32 [2.89 − 3.81]).

**Conclusion:**

After adjustment for potential confounders and PS matching, early relapse was an adverse prognostic factor for survival outcomes in patients with OCSCC. Our findings may have significant implications for risk stratification.

**Supplementary Information:**

The online version contains supplementary material available at 10.1186/s12885-023-10602-1.

## Introduction

Patients with first primary oral cavity squamous cell carcinoma (OCSCC) are generally treated with surgical resection, either with or without adjuvant therapy depending on the presence of post-operative pathological risk factors [1]. According to the National Comprehensive Cancer Network (NCCN) guidelines, OCSCC with extra-nodal extension (ENE) and/or pathologically positive margin(s) are potential candidates for postoperative concurrent chemoradiotherapy (CCRT) [2, 3]. Since the prognosis of patients with OCSCC is heavily dependent on locoregional control, wide excision margins and selective neck dissection (ND; supra-omohyoid ND [level I to III] for cN0 disease or modified ND [level I to V] for cN + disease) are paramount to ensure favorable outcomes [4]. Published studies have consistently shown that OCSCC relapses have an adverse prognostic significance [5–11]. While the use of salvage therapy may improve the clinical outcomes of patients with locoregional relapse, this approach is generally not recommended in presence of distant metastases [6, 12–15] due to their adverse prognostic significance [16]. Distant spread is common (up to 30% of cases) in patients with OCSCC who show regional lymph node metastasis with ENE [17]. Interestingly, a very high rate of distant metastasis (up to 92%) has been reported in a specific subgroup of patients with pN3b disease [18].

The prognostic significance of the time interval between treatment and the first disease relapse in head and neck malignancies remains controversial. Using various cutoff points, previous small-sized studies found that early relapse is an adverse prognostic factor in patients with oral cavity cancer, oropharyngeal cancer, and head and neck cancer [5–10, 19, 20]. More specifically, Kernohan et al. [11] found that the time to recurrence was a predictor of survival in patients with local – but not regional – recurrences. In general, they found that regional recurrences outweighed local recurrences in terms of adverse prognostic significance [11]. The optimal cutoff for the relapse interval in OCSCC has not yet been determined; this may be due, at least in part, by the fact that several previous studies did not include patients who relapsed within certain time frames (e.g., less than 6 months) [7, 9]. In general, patients with late relapse are more likely to receive salvage therapy, resulting in more favorable outcomes.

The aim of this large-scale, registry-based, nationwide study was to investigate whether the time interval between surgery and the first disease relapse may affect survival outcomes in Taiwanese patients with OCSCC. We also examined the prognostic impact of early tumor relapse by taking into account several potential confounding variables – including treatment modalities and traditional clinicopathological risk factors (RFs).

## Methods

### Study setting

The present retrospective study was conducted using data obtained from the Taiwanese Cancer Registry Database (TCRD) “long-form”. The registry has prospectively recorded information on risky oral habits (i.e., preoperative alcohol drinking, betel quid chewing, and cigarette smoking), cancer stage, treatment modalities, and tumor relapses. However, the TCRD did not contain information concerning salvage therapy in patients with relapsing OCSCC. While data on radiation therapy are available, information on chemotherapy regimens are not collected. As of 2011, data on ENE, margin status, and tumor depth of invasion (DOI) were also included. The TCRD collects information from all major Taiwanese hospitals, to which the vast majority (> 99%) of patients with OCSCC are referred. Follow-up data were obtained from the Taiwanese National Health Insurance Research Dataset (TNHIRD). The registry can be openly accessed from university hospitals (Health and Welfare Data Science Center) in Taiwan through the Taiwanese Ministry of Health and Welfare. This study reporting is consistent with the recommendations for tumor marker prognostic studies (REMARK) [21, 22]. All procedures were approved by the Chang Gung Medical Foundation Institutional Review Board (reference number: 201801398B0A3). The requirement for written patient informed consent was waived due to the study design.

### Treatment protocol and follow-up protocol

As part of its continued effort to improve the quality of cancer care, the Taiwan Health Promotion Administration has taken initiative to promote multidisciplinary team care (MDTC) and multidisciplinary case management as of April 2003. Because outcomes in patients with OCSCC are largely dependent on the type of surgical approach and the use of adjuvant therapy, a comprehensive strategy for decision-making, therapy, clinical management, and follow-up is mandatory in betel quid chewing endemic areas. Starting from these premises, all of the Taiwanese hospitals specializing in OCSCC treatment began implementing an MDTC approach as of January 2004. In general, the follow-up protocols were largely in accordance with the NCCN treatment guidelines [3].

### Data collection procedures and identification of tumor relapses

Disease stage was assigned according to the Seventh Edition of the American Joint Committee on Cancer (AJCC) Staging Manual. We subsequently updated the original information in accordance with the AJCC 2018 Staging Manual (Eight Edition) by taking into account both DOI and ENE [23]. The final data analysis was carried out in July 2022 by taking into account the most recent TCRD (2017 release) and TNHIRD (2019 release) data sets. Information on cancer-related morbidity obtained from the TNHIRD was used to calculate disease-specific survival (DSS) and overall survival (OS). While data on local, regional, and distal events are included in the TCRD, their reliability for calculation of disease-free survival (DFS) is questionable. In general, the TCRD complies with the principles outlined by the American College of Surgeons “Standards for Oncology Registry Entry (STORE)”, including information on histology grade [24]. According to the STORE guidelines, data on disease recurrences were collected independently from the anatomical site (i.e., local, regional, or distant); in addition, only the first recurrence was recorded. Information from each hospital was transmitted to the TCRD in the first and fifth years of follow-up. Therefore, survival data were entirely reliable for calculating DSS and OS, although this was not the case for DFS (including the analysis of local control, neck control, and distant metastases). In general, the diagnosis of recurrent disease at follow-up was based on clinical and/or imaging findings. All data managers collected information on disease relapses from pathology and imaging reports. MDTC meeting records were also used as data sources.

### Patient selection

**Fig. 1:**
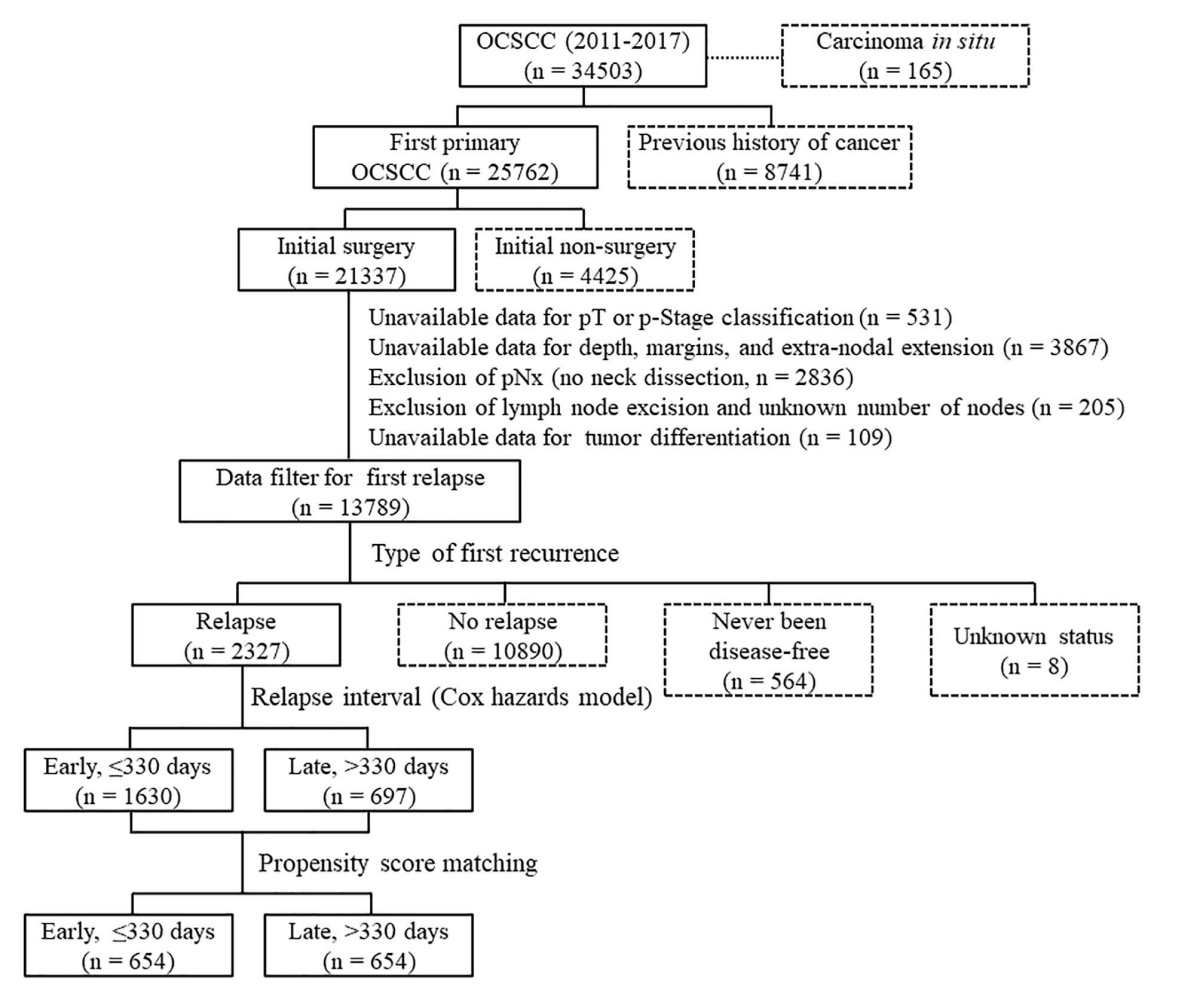
Study flowchart

Patients diagnosed with OCSCC between 2011 and 2017 were eligible for inclusion. Cases were selected according to the following International Classification of Diseases for Oncology, Third Edition [ICDO-3] codes: lip cancer [C00.0; C00.1; C00.2; C00.3; C00.4; C00.5; C00.6; C00.8; C00.9], tongue cancer [C02.0; C02.1; C02.2; C02.3; C02.8; C02.9], alveolar ridge cancer [C03.0; C03.1; C03.9], floor of mouth cancer [C04.0; C04.1; C04.8; C04.9], hard palate cancer [C05.0; C05.8; C05.9], buccal cancer [C06.0], retromolar trigone cancer [C06.2], and other forms of oral cavity cancer [C06.1; C06.8; C06.9]). This patient selection approach is consistent with a previous nationwide study of OCSCC published by our group [25]. Monitoring was continued until December 2019. The study flow chart (Fig. 1) shows details about inclusion and exclusion of cases. Patients were not eligible if the medical records indicated that they (1) had sustained a prior cancer (n = 8741), (2) had initially undergone non-surgical treatment (n = 4425), (3) had an unknown pathological stage (n = 531), (4) had unavailable data concerning DOI, surgical margins, and ENE (n = 3867), (5) had unavailable information on pathological lymph node metastases (n = 2836 + 205), and (6) had no information on tumor differentiation (n = 109). Initially, a total of 13,789 patients were identified. Of them, 564 were never disease-free (TCRD event code: 70) and eight had an unknown recurrence status (code: 99). The final study cohort consisted of 2327 (16.9%) patients with a documented disease relapse. The distribution of the index relapse event (n = 2327) at local (T), neck (N), and distant (M) sites was as follows: T (n = 993, event codes: 13 and 21), N (n = 482, event code: 22), M (n = 452, event codes: 40, 51–58, and 62), T + N (n = 178, event code: 25), T + M or N + M or T + N + M (n = 222, event code: 60). Of the 2327 patients with relapsing disease, 674 (452 + 222, 29.0%) experienced their first relapse as distant metastasis.

### Statistical analysis

The cutoff point for the relapse interval was selected according to two parameters (i.e., timing and first relapse event) and was defined as the breakpoint in the log hazard function. Identification was accomplished by examining the functional relationships between the relapse interval and the hazard ratios for DSS and OS. With this aim, we built a Cox proportional hazards model with a spline function in the R statistical environment. This method for selecting the optimal cutoff point is consistent with a previous nationwide study of OCSCC published by our group [25]. The primary outcome measures were the 5-year DSS and OS rates. The duration of follow-up was defined as the interval from the date of surgery to the date of death. The relapse time was calculated according to the date on which relapsing disease was diagnosed, whereas DSS was determined according to the date on which the relapsing patient died of OCSCC. Censoring was performed on the date of the last follow-up (i.e., administrative censoring). The censoring time was defined by the interval between the date of the index event to the date of last follow-up (December 31, 2019). In our study, all patients were followed-up until December 31, 2019, the only exception being those who experienced the event of interest. Propensity score (PS) matching computed using logistic regression was applied to reduce bias due differences in baseline variables. After PS matching, the clinical outcomes of patients with early *versus* late relapse were compared. We generated Kaplan-Meier plots to estimate survival curves, and differences between the groups were assessed using a log-rank test. To identify significant predictors of survival endpoints, we performed univariate and multivariable Cox proportional hazards regression analyses. A multivariable stepwise selection procedure was applied, and all of the variables included in univariate analysis were entered into the final multivariable model. Since there was a possible violation of the non-informative censoring assumption for DSS, a competing risk model was also implemented. The results were expressed as hazard ratios (HRs) and 95% confidence intervals (CIs). All analyses were carried out in SAS, version 9.4 (SAS Institute Inc., Cary, NC, USA) and R, version 4.0.2 (R Foundation for Statistical Computing, Vienna, Austria). Two-tailed *p* values < 0.05 were considered statistically significant.

## Results

### Optimal cutoff values for the relapse interval

**Fig. 2:**
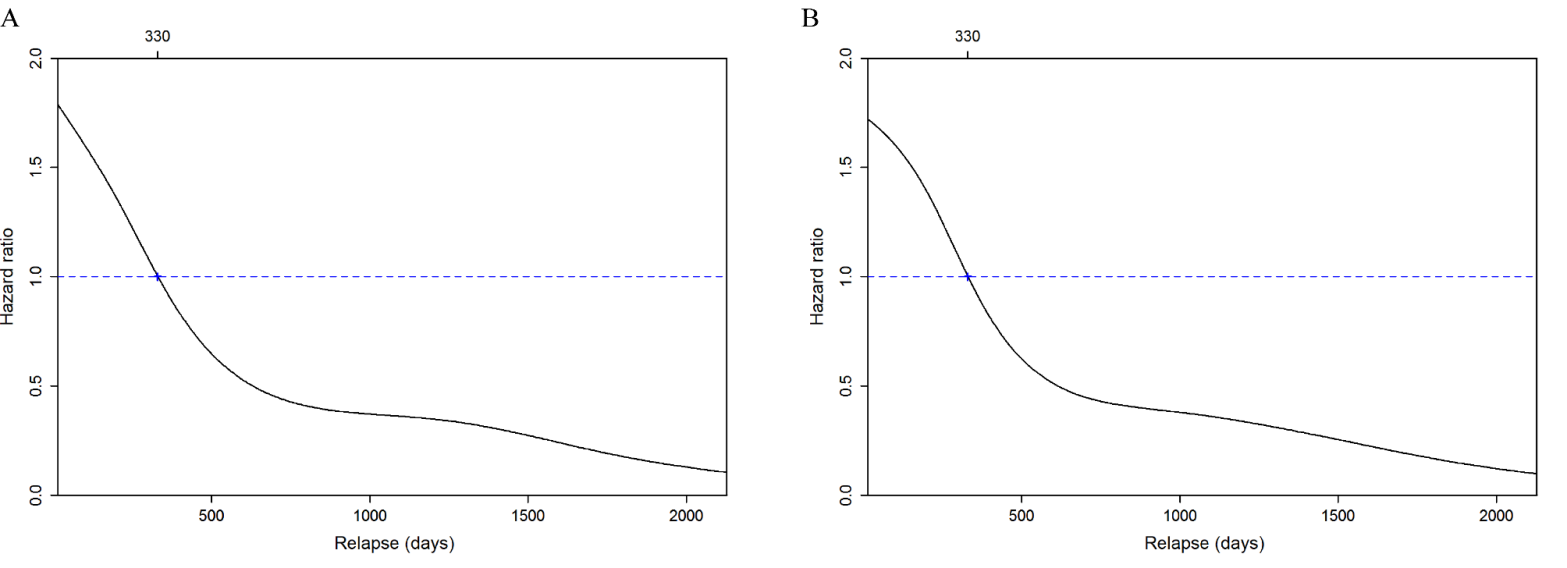
Adjusted hazard ratios for the 5-year disease-specific survival (A) and overall survival (B) according to the relapse interval

The interval from the date of surgery to the date of relapse in patients (n = 2327) who had relapsing disease ranged from 14 to 2128 days (median: 221 days, mean: 387 days, standard deviation: 420 days). Most relapses (73%; 1700/2327) occurred within one year, and 84% of them (1953/2327) were diagnosed within two years. Using the 5-year DSS and OS rates as the outcomes of interest, the results of Cox proportional hazards regression revealed that the optimal cutoff value for the relapse interval was 330 days (early, ≤ 330 days *versus* late, > 330 days, n = 1630/697; Fig. 2A-B).

### General characteristics of patients with early and late relapse

Compared with patients who experienced a late relapse (Table 1), those with an early relapse had a significantly higher prevalence of the following variables (all *p* < 0.0001, the only exception being age): tongue subsite, female sex, age ≥ 65 years, pT4, pN3, pStage IV, poor differentiation, depth ≥ 10 mm, ENE, adjuvant therapy, and distant relapse. Surprisingly, patients with late relapse showed a significantly higher prevalence of positive preoperative smoking history compared with the early relapse group (84% *versus* 79%, respectively, *p* = 0.0115). The early and late relapse groups did not differ significantly in terms of radiotherapy (RT) dose, neck radiotherapy for pN0 disease, and RT techniques (conformal RT *versus* intensity modulated radiation therapy [IMRT] *versus* volumetric-modulated arc therapy [VMAT]). These results were not affected by the use of surgery plus RT *versus* surgery plus chemotherapy plus RT. In addition, we found no significant differences in terms of the interval between surgery and the start of adjuvant therapy in patients who relapsed early compared with the late relapse group. These results were not affected by the use of surgery plus RT *versus* surgery plus chemotherapy plus RT.

**Table 1:** General characteristics of patients with resected first primary oral cavity squamous cell carcinoma according to the relapse interval (early, ≤ 330 days *versus* late, > 330 days) before and after propensity score matching

Characteristic (n)	Original cohort (n = 2327)				Propensity score-matched cohort (n = 1308)			
	Early relapse (n = 1630)	Late relapse (n = 697)	*p* ^*a*^	SMD (%)	Early relapse (n = 654)	Late relapse (n = 654)	*p* ^*b*^	SMD (%)
**Tumor subsite**			< 0.0001				0.9819	
Lip (70)	42 (2.6)	28 (4.0)		-8.07	21 (3.2)	24 (3.7)		-2.52
Tongue (925)	700 (42.9)	225 (32.3)		22.15	232 (35.5)	221 (33.8)		3.54
Gum (295)	212 (13.0)	83 (11.9)		3.33	81 (12.4)	79 (12.1)		0.93
Mouth floor (75)	53 (3.3)	22 (3.2)		0.54	23 (3.5)	22 (3.4)		0.84
Hard palate (35)	28 (1.7)	7 (1.0)		6.16	8 (1.2)	7 (1.1)		1.44
Buccal (742)	473 (29.0)	269 (38.6)		-20.35	234 (35.8)	245 (37.5)		-3.49
Retromolar (117)	77 (4.7)	40 (5.7)		-4.56	33 (5.0)	37 (5.7)		-2.72
Other sites (68)	45 (2.8)	23 (3.3)		-3.15	22 (3.4)	19 (2.7)		2.63
**Sex**			< 0.0001				0.6682	
Men (2141)	1474 (90.4)	667 (95.7)		-20.84	621 (95)	624 (95.4)		-2.14
Women (186)	156 (9.6)	30 (4.3)		20.84	33 (5)	30 (4.6)		2.14
**Age (years)**			0.0204				0.9244	
< 65 (2015)	1394 (85.5)	621 (89.1)		-10.75	581 (88.8)	580 (88.7)		0.48
≥ 65 (312)	236 (14.5)	76 (10.9)		10.75	73 (11.2)	74 (11.3)		-0.48
**Alcohol drinking**			0.2019				0.5090	
No (730)	511 (31.3)	219 (31.4)		-0.15	204 (31.2)	206 (31.5)		-0.66
Yes (1586)	1114 (68.3)	472 (67.7)		1.34	448 (68.5)	443 (67.7)		1.64
Missing data (11)	5 (0.4)	6 (0.9)		-7.28	2 (0.3)	5 (6.8)		-6.29
**Betel quid chewing**			0.2523				0.8145	
No (595)	432 (26.5)	163 (23.4)		7.21	156 (23.9)	158 (24.2)		-0.72
Yes (1716)	1186 (72.8)	530 (76.0)		-7.52	492 (75.2)	492 (75.2)		0.00
Missing data (16)	12 (0.7)	4 (0.6)		2.01	6 (0.9)	4 (0.6)		3.51
**Cigarette smoking**			0.0115				0.6065	
No (447)	337 (20.7)	110 (15.8)		12.70	104 (15.9)	104 (15.9)		0.00
Yes (1877)	1290 (79.1)	587 (84.2)		-13.15	549 (83.9)	550 (84.1)		-0.42
Missing data (3)	3 (0.2)	0 (0.0)		6.07	1 (0.2)	0 (0.0)		5.53
**Pathologic T status**			< 0.0001				0.8647	
T1 (299)	146 (9.0)	153 (22.0)		-36.54	121 (18.5)	123 (18.8)		-0.79
T2 (641)	410 (25.2)	231 (33.1)		-17.65	217 (33.2)	222 (33.9)		-1.62
T3 (402)	294 (18.0)	108 (15.5)		6.81	117 (17.9)	106 (16.2)		4.47
T4 (985)	780 (47.8)	205 (29.4)		38.57	199 (30.4)	203 (31.1)		-1.33
**Pathologic N status**			< 0.0001				0.8308	
pN0 (1107)	672 (41.2)	435 (62.4)		-43.38	388 (59.3)	397 (60.7)		-2.81
pN1 (258)	185 (11.3)	73 (10.5)		2.81	75 (11.5)	72 (11.0)		1.45
pN2 (379)	272 (16.7)	107 (15.4)		3.64	101 (15.4)	103 (15.7)		-0.84
pN3 (583)	501 (30.8)	82 (11.7)		47.68	90 (13.8)	82 (12.6)		3.62
**Pathologic stage**			< 0.0001				0.8650	
I (236)	110 (6.7)	126 (18.1)		-34.88	96 (14.7)	97 (14.8)		-0.43
II (368)	225 (13.8)	143 (20.5)		-17.88	127 (19.4)	137 (20.9)		-3.81
III (339)	224 (13.7)	115 (16.5)		-7.7	119 (18.2)	112 (17.1)		3.00
IV (1384)	1071 (65.8)	313 (44.9)		42.78	312 (47.7)	308 (47.2)		2.81
**Tumor differentiation**			< 0.0001				0.7250	
Well (445)	257 (15.8)	188 (27.0)		-27.6	168 (25.7)	163 (24.9)		1.76
Moderately (1536)	1089 (66.8)	447 (64.1)		5.63	417 (63.8)	429 (65.6)		-3.84
Poorly (344)	282 (17.3)	62 (8.9)		25.11	69 (10.5)	62 (9.5)		3.57
Undifferentiated (2)	2 (0.1)	0 (0.0)		4.96	0 (0.0)	0 (0.0)		0.00
**Depth of invasion**			< 0.0001				0.7355	
< 10 mm (933)	546 (33.5)	387 (55.5)		-45.45	340 (52.0)	345 (52.8)		-1.53
≥ 10 mm (1394)	1084 (66.5)	310 (44.5)		45.45	314 (48.0)	309 (47.2)		1.53
**Margin status**			0.0902				0.9524	
< 5 mm (1304)	932 (57.2)	372 (53.4)		7.66	356 (54.4)	357 (54.6)		-0.31
≥ 5 mm (1023)	698 (42.8)	325 (46.6)		-7.66	298 (45.6)	297 (45.4)		0.31
**Extranodal extension**			< 0.0001				0.6042	
No (1641)	1055 (64.7)	586 (84.1)		-45.47	538 (82.3)	544 (83.2)		-2.43
Yes (686)	575 (35.3)	111 (15.9)		45.47	116 (17.7)	110 (16.8)		2.43
**Treatment modality**			< 0.0001				0.2414	
S alone (841)	541 (33.2)	300 (43.0)		-20.39	252 (38.5)	271 (41.4)		-5.93
S plus CT + S plus RT + S plus CT and RT (1486)	1089 (66.8)	397 (57.0)		20.39	402 (61.5)	383 (58.6)		5.93
**Distant relapse**			< 0.0001				0.8563	
No (1653)	1064 (65.3)	589 (84.5)		-45.47	548 (83.8)	546 (83.5)		0.83
Yes (674)	566 (34.7)	108 (15.5)		45.47	106 (16.2)	108 (16.5)		-0.83
**Time interval (days)**								
Range	14–330	331–2128						
Median	173	775						
Mean	174	886						
**RT technique in the S + RT subgroup (n = 342)**			0.1996					
Conformal (10)	6 (2.6)	4 (3.5)						
IMRT (169)	105 (46.3)	64 (55.7)						
VMAT (163)	116 (51.1)	47 (40.9)						
**RT dose (cGy) in the S + RT subgroup (n = 342)**								
Range (200–8200)	200–8200	1000–7050						
Median (6000)	6000	6050	0.8214^c^					
Mean (5959.9)	5875.0	6127.4						
**S-RT interval in the S + RTsubgroup (n = 342)**								
Range (14–146 days)	14–146	15–79						
Median (37 days)	37	37	0.5410^c^					
Mean (38.6 days)	39.3	37.1						
**RT Technique in the S + CT + RT subgroup** **(n = 1071)**			0.0537					
Conformal (17)	15 (1.9)	2 (0.8)						
IMRT (531)	384 (47.6)	147 (55.5)						
VMAT (523)	407 (50.5)	116 (43.8)						
**RT dose (cGy) in the S + CT + RT subgroup** **(n = 1071)**								
Range (600–8700)	600–7800	600–8700						
Median (6600)	6600	6600	0.3324^c^					
Mean (6414.0)	6403.2	6446.6						
**S-RT interval in the S + CT + RT subgroup** **(n = 1071)**								
Range (13–211 days)	14–211	13–162						
Median (38 days)	38	37	0.1593^c^					
Mean (40.7 days)	40.5	41.5						
**RT field in pN0 (n = 378)**			0.9584					
Primary tumor + neck (106)	65 (28.1)	41 (27.9)						
Primary tumor only (272)	166 (71.9)	106 (72.1)						

*SMD* standardized mean difference, *S* surgery, *CT* chemotherapy, *RT* radiotherapy, *IMRT* intensity modulated radiation therapy, *VMAT* Volumetric-modulated arc therapy

^*a*^ Chi-square test; ^*b*^ McNemar test; ^c^ Mann-Whitney *U* Test (skewed data)

### Relapse site stratified according to the occurrence of early ***versus*** late relapse

Table 2 shows the site of relapse (i.e., local, neck, and distant) stratified according to the occurrence of early *versus* late relapse. A highly significant difference (*p* < 0.0001) was observed, especially with respect to the more frequent occurrence of local relapse in the late relapse group.

**Table 2:** Patterns of disease relapse in the early *versus* late relapse groups

	Entire cohort of patients with relapsing disease (n = 2327)	Early relapse	Late relapse	*p*
		(n = 1630)	(n = 697)	
Site of recurrence				< 0.0001
T	993 (42.7)	522 (32.0)	471 (67.6)	
N	482 (20.7)	405 (24.8)	77 (11.0)	
M	452 (19.4)	367 (22.5)	85 (12.2)	
T + N	178 (7.6)	137 (8.4)	41 (5.9)	
T + M or N + M or T + N + M	222 (9.5)	199 (12.3)	23 (3.3)	

*T* local relapse, *N* neck relapse, *M* distant relapse

### Five-year survival rates

**Fig. 3:**
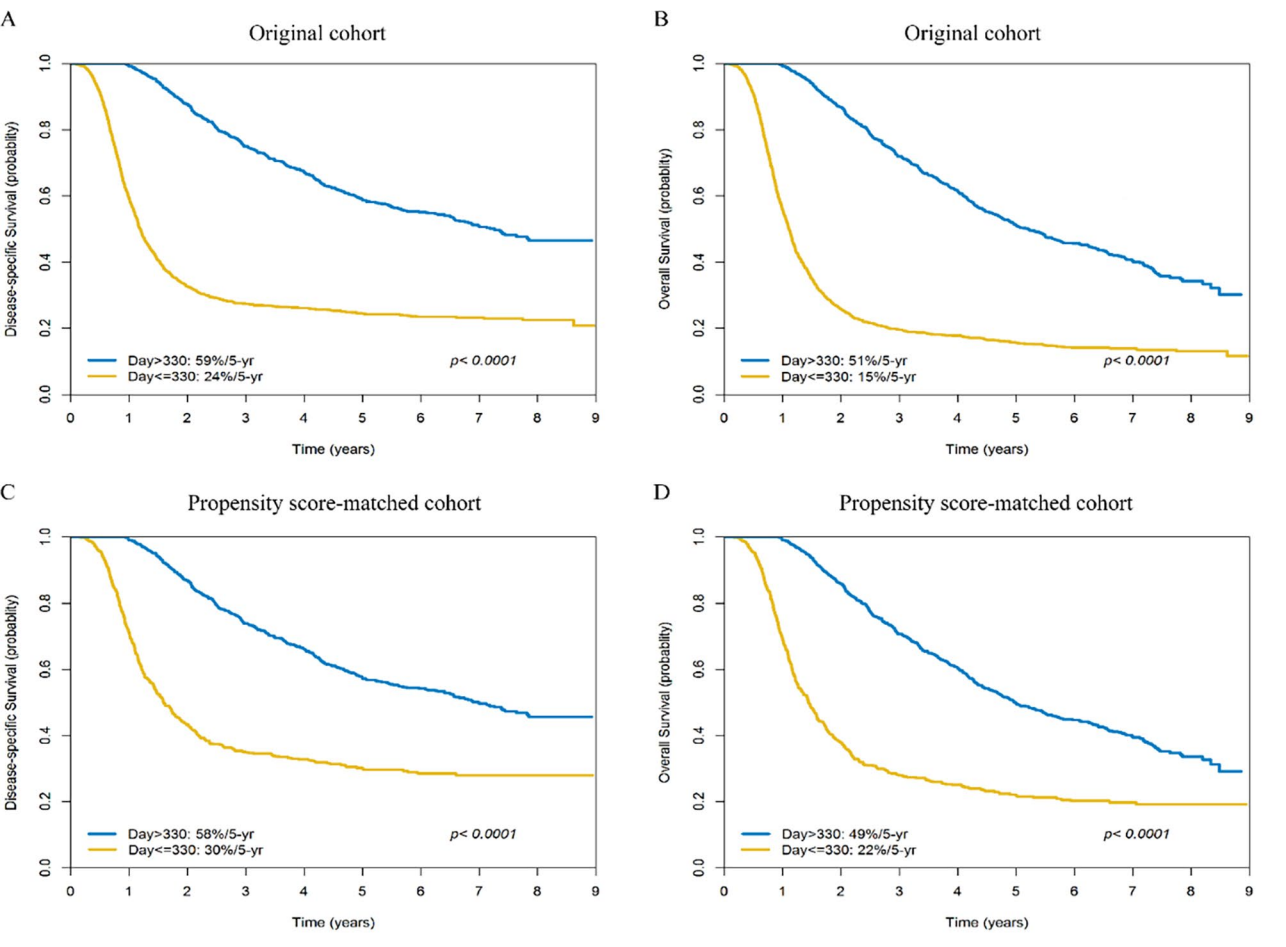
Kaplan-Meier plots of disease-specific survival (panel A) and overall survival (panel B) in patients with early (≤ 330 days) *versus* late (> 330 days) relapse in the original cohort (n = 2327). Kaplan-Meier plots of disease-specific survival (panel C) and overall survival (panel D) in patients with early (≤ 330 days) *versus* late (> 330 days) relapse in the propensity score-matched cohort (n = 1308)

The 5-year DSS/OS rates observed in the original cohort of 13,789 patients were 77%/69%, respectively. The 5-year DSS and OS rates of patients without relapsing disease (n = 10,890) *versus* those of patients with relapsing disease (n = 2327) were 89% *versus* 34% (*p* < 0.0001) and 82% *versus* 26% (*p* < 0.0001), respectively. The 5-year DSS and OS rates of patients with late *versus* early relapse were 59% *versus* 24% (*p* < 0.0001) and 51% *versus* 15% (*p* < 0.0001), respectively (Fig. 3A-B).

The median follow-up time in the entire cohort (n = 2327) was 702 days (mean: 1101 days; standard deviation: 928 days, range: 5 − 3274 days). In the early relapse group (n = 1630), the median duration of follow-up was 433 days (mean: 774 days; standard deviation: 862 days, range: 5 − 3274 days). Finally, the median follow-up time in the late relapse group (n = 697) was 2001 days (mean: 1865 days; standard deviation: 729 days, range: 344 − 3260 days).

### Univariate and multivariable weighted Cox regression analysis (average hazard ratio, AHR)

The results of standard Cox proportional hazards regression analysis (HR) are presented in Supplementary Table 1. Notably, the findings obtained from competing risk model analysis of DSS were in line with those of Cox proportional hazards regression analysis (early relapse, [subdistribution hazard ratio, SHR: 2.50, *p* < 0.0001]) (Supplementary Table 1).

The plot of log(-log(survival) *versus* time showed a mild violation of the proportional hazard assumption. We therefore resorted to a weighted Cox regression (average hazard ratio, AHR) as a robust alternative to the standard Cox estimator [26]. Univariate analyses identified the following parameters as significantly associated with less favorable 5-year survival rates; DSS: early relapse (AHR: 4.05, *p* < 0.0001), tongue/gum subsites, age ≥ 65 years, pT2 − 4, pN1 − 3, pStage II − IV, absence of a well-differentiated tumor, DOI ≥ 10 mm, ENE, adjuvant therapy, and distant relapse; OS: early relapse (AHR: 4.70, *p* < 0.0001), hard palate subsite, age ≥ 65 years, pT2 − 4, pN1 − 3, pStage II − IV, absence of a well-differentiated tumor, DOI ≥ 10 mm, margin < 5 mm, ENE, adjuvant therapy, and distant relapse (Table 3).

**Table 3:** Univariable and multivariable analyses of risk factors for 5-year disease-specific and overall survival in the entire study cohort (n = 2327) according to average hazard ratio (AHR, weighted Cox regression) using the day of surgery as the index date

Risk factor	Disease-specific survival				Overall survival			
	Univariable analysis^a^		Stepwise multivariable analysis^a^		Univariable analysis^a^		Stepwise multivariable analysis^a^	
	AHR (95% CI)	*p*	AHR (95% CI)	*p*	AHR (95% CI)	*p*	AHR (95% CI)	*p*
**Relapse interval**								
Late (> 330 days)	1		1		1		1	
Early (≤ 330 days)	4.05 (3.63–4.51)	< 0.0001	3.24 (2.90–3.62)	< 0.0001	4.70 (4.24–5.20)	< 0.0001	3.91 (3.51–4.35)	< 0.0001
**Tumor subsite**								
Lip	1		1		1		1	
Tongue	1.46 (1.05–2.04)	0.0254	-	ns	1.25 (0.93–1.69)	0.1363	-	ns
Gum	1.52 (1.07–2.16)	0.0189	-	ns	1.32 (0.96–1.81)	0.0885	-	ns
Mouth floor	1.32 (0.88–1.99)	0.1819	-	ns	1.26 (0.87–1.83)	0.2199	-	ns
Hard palate	1.53 (0.93–2.50)	0.0942	-	ns	1.56 (1.01–2.41)	0.0443	-	ns
Buccal	1.23 (0.88–1.72)	0.2204	-	ns	1.07 (0.79–1.45)	0.6544	-	ns
Retromolar	1.26 (0.84–1.87)	0.2604	-	ns	1.18 (0.82–1.68)	0.3698	-	ns
Other sites	1.03 (0.64–1.64)	0.9160	-	ns	1.13 (0.74–1.72)	0.574	-	ns
**Sex**								
Men	0.92 (0.76–1.12)	0.3968	-	ns	0.93 (0.77–1.13)	0.4785	1.24 (1.02–1.52)	0.0297
Women	1		1		1		1	
**Age (years)**								
< 65	1		1		1		1	
≥ 65	1.19 (1.02–1.39)	0.0258	1.22 (1.03–1.44)	0.0197	1.24 (1.08–1.43)	0.0026	1.32 (1.13–1.54)	0.0004
**Alcohol drinking**								
No	1		1		1		1	
Yes	1.07 (0.96–1.20)	0.2129	-	ns	1.08 (0.97–1.20)	0.1703	-	ns
Missing information	0.45 (0.15–1.34)	0.1502	-	ns	0.50 (0.18–1.08)	0.1802	-	ns
**Betel quid chewing**								
No	1		1		1		1	
Yes	1.07 (0.95–1.20)	0.2967	-	ns	1.08 (0.96–1.21)	0.1900	-	ns
Missing information	1.08 (0.57–2.06)	0.8074	-	ns	1.23 (0.74–2.06)	0.4168	-	ns
**Cigarette smoking**								
No	1		1		1		1	
Yes	0.93 (0.82–1.07)	0.3127	-	ns	0.97 (0.85–1.10)	0.5898	-	ns
Missing information	1.26 (0.33–4.75)	0.7366	-	ns	1.14 (0.29–4.41)	0.8506	-	ns
**Pathologic T status**								
T1	1		1		1		1	
T2	1.90 (1.56–2.33)	< 0.0001	-	ns	1.93 (1.60–2.34)	< 0.0001	1.45 (1.17–1.79)	0.0006
T3	2.80 (2.27–3.44)	< 0.0001	-	ns	2.92 (2.39–3.55)	< 0.0001	1.58 (1.20–2.08)	0.0012
T4	4.16 (3.43–5.03)	< 0.0001	-	ns	4.59 (3.84–5.49)	< 0.0001	1.95 (1.51–2.51)	< 0.0001
**Pathologic N status**								
pN0	1		1		1		1	
pN1	1.91 (1.61–2.26)	< 0.0001	1.23 (0.99–1.53)	0.0670	1.86 (1.58–2.19)	< 0.0001	1.38 (1.14–1.66)	0.0009
pN2	2.44 (2.10–2.83)	< 0.0001	1.43 (1.17–1.74)	0.0004	2.36 (2.06–2.71)	< 0.0001	1.64 (1.41–1.92)	< 0.0001
pN3	3.77 (3.31–4.30)	< 0.0001	1.79 (1.48–2.15)	< 0.0001	4.05 (3.56–4.60)	< 0.0001	2.26 (1.94–2.63)	< 0.0001
**Pathologic stage**								
I	1		1		1		1	
II	1.51 (1.17–1.94)	< 0.0001	1.24 (0.95–1.62)	0.1120	1.57 (1.24–1.98)	0.0001	-	ns
III	2.58 (2.03–3.29)	< 0.0001	1.71 (1.27–2.30)	0.0004	2.53 (2.01–3.17)	< 0.0001	-	ns
IV	5.14 (4.14–6.38)	< 0.0001	2.10 (1.57–2.80)	< 0.0001	5.44 (4.46–6.64)	< 0.0001	-	ns
**Tumor differentiation**								
Well	1		1		1		1	
Moderately	1.41 (1.23–1.61)	< 0.0001	-	ns	1.41 (1.25–1.60)	< 0.0001	1.06 (0.92–1.22)	0.4426
Poorly	2.11 (1.77–2.52)	< 0.0001	-	ns	2.25 (1.91–2.66)	< 0.0001	1.36 (1.14–1.62)	0.0006
Undifferentiated	8.47 (1.68–42.65)	< 0.0001	-	ns	7.79 (1.51–40.15)	< 0.0001	1.41 (0.27–7.34)	0.6838
**Depth of invasion**								
< 10 mm	1		1		1		1	
≥ 10 mm	2.25 (2.01–2.52)	< 0.0001	1.92 (1.68–2.20)	0.0153	2.35 (2.11–2.61)	< 0.0001	1.19 (1.001–1.42)	0.0483
**Margin status**								
< 5 mm	1.11 (0.997–1.23)	0.0560	-	ns	1.11 (1.01–1.23)	0.034	-	ns
≥ 5 mm	1		1		1		1	
**Extra-nodal extension**								
No	1		1		1		1	
Yes	2.55 (2.27–2.85)	< 0.0001	-	ns	2.74 (2.46–3.05)	< 0.0001	-	ns
**Treatment modality**								
S alone	1		1		1		1	
S plus CT + S plus RT + S plus CT and RT	2.29 (2.04–2.58)	< 0.0001	-	ns	2.28 (2.05–2.55)	< 0.0001	-	ns
**Distant relapse**								
No	1		1		1		1	
Yes	3.16 (2.82–3.54)	< 0.0001	1.92 (1.68–2.20)	< 0.0001	3.63 (3.25–4.06)	< 0.0001	2.30 (2.02–2.63)	< 0.0001

*AHR* average hazard ratio, *CI* confidence interval, *ns* not significant, *S* surgery, *CT* chemotherapy, *RT* radiotherapy

^a^ In univariable analyses, each covariate was added separately to the Cox regression model. In multivariable analysis, all covariates were entered into the Cox regression using a stepwise procedure for variable selection. The symbol “-” indicates that the corresponding variable was excluded from the Cox regression model after applying the stepwise selection procedure

Multivariable analyses identified the following RFs as independently associated with less favorable 5-year survival rates; DSS: early relapse (AHR: 3.24, *p* < 0.0001), age ≥ 65 years, pN2 − 3, p-Stage III-IV, DOI ≥ 10 mm, and distant relapse; OS: early relapse (AHR: 3.91, *p* < 0.0001), male sex, age ≥ 65 years, pT2-T4, pN1 − 3, poor tumor differentiation, DOI ≥ 10 mm, and distant relapse (Table 3).

### Subgroup analyses of patients with early ***versus*** late relapses after propensity score matching

Since patients with early relapse had more severe disease compared to those with late relapse, PS matching was applied to account for these differences. Two PS-matched groups (n = 654 each) of patients with early *versus* late relapse were identified (Table 1). The results of PS-matched analysis revealed that – compared to patients with late relapse – those with early relapse showed less favorable 5-year DSS: 58% *versus* 30%, *p* < 0.0001 (AHR: 3.10 [2.69–3.57]) and OS: 49% *versus* 22%, *p* < 0.0001 (AHR: 3.32 [2.89–3.81]) (Fig. 3C-D).

### Five-year survival rates after setting the relapse time as point 0

**Fig. 4:**
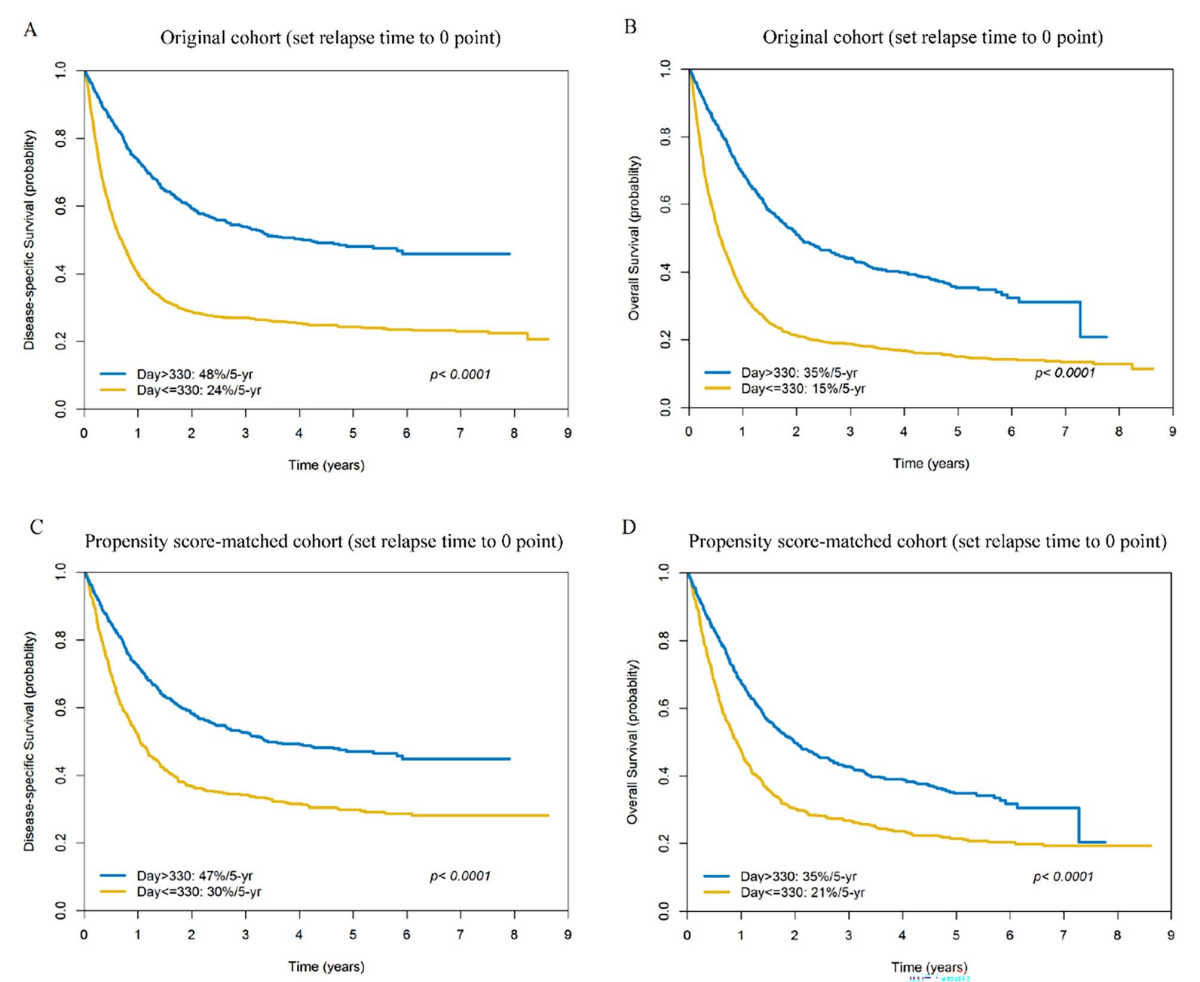
Kaplan-Meier plots of disease-specific survival (panel A) and overall survival (panel B) in patients with early (≤ 330 days) *versus* late (> 330 days) relapse in the original cohort (n = 2327) after setting the relapse time as point 0. Kaplan-Meier plots of disease-specific survival (panel C) and overall survival (panel D) in patients with early (≤ 330 days) versus late (> 330 days) relapse in the propensity score-matched cohort (n = 1308) after setting the relapse time as point 0

In order to account for the immortal time bias – which should be considered if exposure status is determined based on a measurement or event that occurs after baseline – we redefined the time of relapse as “point 0”. When this approach was applied, the 5-year DSS/OS rates of patients with late *versus* early relapse were 48%/24% (*p* < 0.0001) and 35%/15% (*p* < 0.0001), respectively (Fig. 4A-B).

### Univariable and multivariable weighted cox regression analysis after setting the relapse time as point 0

When the index date was calculated from the day of relapse, the results of univariable and multivariable weighted Cox regression analysis were largely consistent with those obtained from the original data set (DSS, AHR: 1.81 [1.59 − 2.05], *p* < 0.0001; OS, AHR: 1.78 (1.57 − 2.01), *p* < 0.0001; Supplementary Table 2).

### Subgroup analyses in the propensity score-matched cohort after setting the relapse time as point 0

When the index date was calculated from the day of relapse, the results obtained in the PS-matched cohort revealed that – compared with patients who showed late relapse – those with early relapse had less favorable 5-year DSS rates (AHR: 1.80 [1.56 − 2.09]; 47%/30%, *p* < 0.0001) and OS (AHR: 1.67 [1.45 − 1.92]; 35%/21%, *p* < 0.0001; (Fig. 4C-D).

## Discussion

Early recurrence is an adverse prognostic marker in patients with head and neck SCC [27]. However, the question as to whether the same applies to all malignancies of the head and neck area remains unanswered. First, tumors located at certain subsites (e.g., oropharynx and hypopharynx) rarely receive initial surgical treatment; in this scenario, only clinical staging can be implemented. Second, the correct endpoint for patients initially treated without surgery should be progression-free survival rather than recurrence-free survival. This is, to our knowledge, the first nationwide cohort study to investigate the impact of the relapse interval on the survival outcomes of patients with OCSCC after curative resection. Compared with patients with late relapse (> 330 days), those who relapsed early (≤ 330 days) had less favorable 5-year DSS and OS rates. After adjustment for potential confounders, early relapse was retained in the multivariable model as an independent adverse prognostic factor. Additionally, the adjusted AHRs for early relapse (3.24 for 5-year DSS and 3.91 for 5-year OS) were the highest among all of the independent RFs.

Since patients who relapsed early had a higher prevalence of several adverse prognostic markers compared with the late relapse group, PS matching was applied to minimize their potential confounding effect. The results – which were largely in accordance with those obtained from the original cohort – confirmed an adverse prognostic significance of early relapse for both survival endpoints (AHR for 5-year DSS: 3.10; AHR for 5-year OS: 3.32). Collectively, these data indicate that the occurrence of early relapse outweighed the prognostic impact of both traditional clinicopathological RFs and treatment modalities.

Based on these findings, patients who showed late relapses were expected to have more favorable survival outcomes than those who showed an early relapse. We therefore performed additional analyses to rule out the potential impact of an immortal time bias. This was accomplished by considering the day of relapse as the index date. The results were entirely consistent with those obtained in the original cohort – despite a decline of AHRs being evident in the early relapse group (PS-matched analysis, DSS: AHR from 3.10 to 1.80; OS: AHR from 3.32 to 1.67).

In the current study, pT4, pN3, Stage IV, poor differentiation, depth ≥ 10 mm, ENE, and distal relapse were associated with early relapse. These adverse prognostic factors are in accordance with those described in the published literature. Notably, it has been previously shown that early relapsing tumors exhibit a more aggressive biological behavior and are characterized by higher maximum standardized uptake values on FDG-PET images [18].

In the original cohort, the early relapse group included a higher percentage of women compared with late relapse group (9.6% *versus* 4.3%, respectively, *p* < 0.0001, Table 1). Further analyses in relation to various clinicopathological RFs revealed no sex-related differences with respect to the following variables: pT status, pN status, p-Stage, DOI, margin status, treatment modality, and distant relapse. However, compared with men, women showed a higher number of tumors located in the tongue (61% *versus* 38%, *p* < 0.0001), age ≥ 65 years (31% *versus* 12%, *p* < 0.0001), poorly differentiated tumors (23% *versus* 14%, *p* = 0.0017), and ENE (37% *versus* 29%, *p* = 0.0273). Since betel nut chewing is endemic in our country, OCSCC is highly common. Notably, Taiwanese women with OCSCC rarely have a positive history of betel nut chewing. In this scenario, the male-to-female ratio for OCSCC in Taiwan is approximately 10-to-1 [28]. The higher prevalence of women in the early recurrence group may be explained by a preponderance of female patients harboring poorly differentiated tumors and ENE – both being RFs related to tumor recurrence [29].

Smoking is a well-known risk factor for OCSCC. Of the five published studies focusing on clinical outcomes of patients with relapsing OCSCC (Table 4), only one conducted by our group (Liao et al., 1996 − 2005) specifically focused on the effect of smoking. In our previous study [5], we found a trend towards a higher prevalence of preoperative smoking history in patients who relapsed early compared with the late relapse group (89% [143/161] *versus* 83% [92/111], respectively, *p* = 0.160). Conversely, in the current study, patients with late relapse showed a significantly higher prevalence of positive preoperative smoking history compared with the early relapse group (84% *versus* 79%, respectively, *p* = 0.0115, Table 1). The reasons underlying these discrepancies are unclear. However, smoking was not a significant risk factor for 5-year DSS and OS in patients with relapsing OCSCC both in our previous study [5] and in the current investigation. The question as to whether the association between late relapse and smoking can be ascribed to the development of recurrent disease *versus* second primary tumors merits comment. In this regard, it is noteworthy that patients in the late relapse group more commonly showed local recurrence compared with regional recurrence (Table 2). However, the results of this analysis should be interpreted cautiously because only the first recurrence event was collected in the TCRD registry. Consequently, the occurrence of neck relapse can be severely underestimated. Surgery remains the mainstay of treatment for first primary and recurrent OCSCC. However, the role of salvage surgery in patients who relapse early but do not harbor distant metastases is a matter of ongoing debate. We have previously shown that – in patients with OCSCC who relapsed early (optimal cutoff for relapse time: 10 months) – no survival differences were evident between salvage RT/CCRT and salvage surgery [5]. However, in the late relapse group, treatment with surgery was associated with better outcomes compared with RT/CCRT [5].

**Table 4:** Published studies focusing on the clinical outcomes of patients with relapsing oral cavity cancer according to the relapse interval

Authors (year of recruitment)	Relapse % (relapsed patients/original cohort), relapse by year	Exclusion based on the relapse interval	p-Stage I − II/III − IV (%)	Method used for determining the cutoff for the relapse interval	Definition of early/late relapse (number of patients)	Risk factors for survival outcomes (MVA)	Five-year survival rate (early/late relapse)
Liao et al. (current study) (2011 − 2017)	16.9% (2327/13,789), 84%/2-year	None	26%/74%	Cox proportional hazards model with spline	≤ 330 days (1630) />330 days (697). After propensity score matching: ≤ 330 days (654) />330 days (654)	DSS: relapse interval, age, pN, p-Stage, DOI, distant relapse OS: relapse interval, male sex, age, pT, pN, poor differentiation, DOI, distant relapse	Propensity score- matched groups: DSS: 30%/58% (*p* < 0.0001); OS: 22%/49% (*p* < 0.0001)
Liao et al. [5] (1996 − 2005)	28.5% (272/953) NR	None	28%/72%	Kaplan-Meier	≤ 10 months (161) />10 months (111)	Early relapse (DSS/OS) ^a^: p-Stage, poor differentiation, DOI, distant relapse Late relapse (DSS/OS) ^a^: pT, poor differentiation, ENE, distant relapse, neck recurrence	DSS: 14%/54% (*p* < 0.0001), OS: 12%/54% (*p* < 0.0001)
Weckx et al. [6] (2002 − 2015)	23% (159/691) 60%/2-year	< 6 weeks	43%/57%	Every 4 months followed by 1 year	1–4 months (11) /5–8 months (32) /9–12 months (15) /13–24 months (37) />24 months (64)	OS: relapse interval, ENE, margins, salvage therapy	OS: 0%/40%/52%/49% /87% (*p* < 0.001)
Liu et al. [7] (1995 − 2003)	50.5% (648/1282) NR	< 6 months	54%/46%	ROC curve analysis	< 18 months (239) /≥18 months (162)	OS: relapse interval, age, p-Stage	Survival after relapse: 28%/38% (*p* < 0.001)
Schwartz et al. [8] (1956 − 1992)	28% (99/350)^b^ 92%/3-year	≤ 3 months	47%/53%	NR	≤ 6 months (11) />6 months (27)	NR	Mean survival time: 20/54 months (*p*, NR)
Mucke et al. [9] (1992 − 2006)	23.9% (185/773) 71%/3-year	≤ 6 months	46%/54%	ROC curve analysis	≤ 18 months (88) />18 months (97)	NR	Survival after relapse: 21%/42% (*p* = 0.03)

*MVA* multivariable analysis, *DSS* disease-specific survival, *OS* overall survival, *ROC* receiver operating characteristic, *DOI* depth of invasion, *ENE* extra-nodal extension, *NR* not reported

^a^ Identical prognostic factors for DSS and OS; ^b^ Thirty-eight of 99 patients met the inclusion criteria

Another investigation reported that stage I − II primary tumors are generally candidates for salvage surgery, regardless of their initial treatment [8]. In our previous study, we found that patients with late relapse may benefit from salvage therapy, especially in presence of local recurrences. In the early relapse group, salvage therapy should be considered for cases with a primary tumor depth < 10 mm [5].

On univariate and multivariable analyses, Kernohan and coworkers [11] found that the initial use of a combined treatment (i.e., surgery plus adjuvant therapy) was associated with less favorable outcomes compared with monotherapies (i.e., surgery only or radiotherapy only). The results of our study indicated surgery plus adjuvant therapy (*versus* surgery alone) was a significant risk factor for 5-year DSS and OS in univariate analysis; however, this association did not persist after adjustment for potential confounders in multivariable analysis. This can be attributed to the high collinearity with other risk factors, including pN3 disease and early relapse (Table 3). In light of the lower survival rate observed in patients who received adjuvant therapy (*versus* those treated with surgery alone), we speculate that the use of salvage therapy in the adjuvant group would be lower than that in patients who received surgery as monotherapy. Unfortunately, the TCRD registry does not contain data on salvage treatment.

The clinical usefulness of postoperative adjuvant therapy in the early *versus* late relapse groups is another point that merits consideration. In general, the potential therapeutic benefits of adjuvant RT have to be critically weighted against the detrimental effects it might cause. Because the time to relapse has prognostic significance, as our study shows, we took this variable into account when devising our therapeutic policies. The use of aggressive adjuvant RT/CCRT schemes is clinically feasible for patients with early relapsing tumors and a negative history of previous head and neck irradiation who have the potential to be cured. In an effort to achieve complete eradication of occult cancer cells, the radiation field we use for these patients comprises both the primary tumor bed and neck lymphatics. For example, when an early isolated nodal relapse is identified, high-dose radiation is delivered to the grossly involved nodal area and the primary tumor bed. However, in the event of a late isolated nodal relapse, the decision to include or exclude the previous primary tumor bed from the prophylactic radiation dose should be guided by a comprehensive assessment of the patient’s clinicopathological parameters, expectations, general conditions, and family support. Special attention must be paid to high-risk patients who have evidence of positive margins, ENE, or multiple pathological risk factors. In this scenario, FDG-PET/CT imaging during the pre-adjuvant phase or CT simulation exams may show evidence of tumor re-growth. Under these circumstances, delivery of RT to gross disease may require either additional adjustments to the radiation field or dose-escalation. As for patients with early relapsing tumors and a positive history of head and neck irradiation, it is our policy to consider whether the target area overlaps for more than 90% with the previous high-dose area. If this is the case, re-irradiation is not implemented due to the potential risk of severe morbidity. In addition, when lesion recurrence occurs shortly after radiotherapy, a dismal outcome is to be expected. This is due to the presence of radioresistant tumors not amenable to aggressive re-irradiation; in this scenario, supportive care represents the most suitable choice. However, advanced RT techniques may be applied when re-irradiation of different head and neck locations is required. In the event of late relapsing disease, standard RT techniques are generally sufficient to achieve adequate tumor control; however, some modifications may be required in the event of pre-existing head and neck irradiations.

Herein, the 5-year OS rates of patients with and without relapsing tumors were 26% and 82%, respectively. In the former group, the 5-year OS rate was less favorable in presence of early relapse (15% *versus* 51% in the late relapse group). These findings are in accordance with those reported in our previous study [5]. After excision of the primary tumor with curative intent, the relapse rates of patients with OCSCC range between 23% and 51% (recurrent events within the first two years: 60 − 92%) [5–9, 11, 13, 30]. In the current study, the relapse rate was as low as 17%; additionally, 73% and 84% of all relapse events occurred within one and two years, respectively (Table 4). While the relapse rate in this study was lower than those reported in the published literature, the temporal distribution of the events was consistent with previous findings. Patients with higher p-Stage, a higher frequency of positive and close margins, and long-term follow-up performed at regular intervals may have higher relapse rates. While p-Stage III − IV disease was highly common in our cohort (74%), the low relapse rate (17%) may be explained by the irregular follow-up schedule (Table 4) – which was dependent on the nature of the data set. However, the large sample size may have partially offset this issue.

A relatively limited number of studies have focused on the prognostic significance of the relapse interval in patients with OCSCC [5–11]. Most of them included patients with relapsing disease who were not stratified according to the treatment modality [5–9]; however, two studies were specifically carried out in patients who underwent salvage therapy [10, 11]. On analyzing the relapse interval, some studies excluded patients who relapsed within certain time frames (e.g., from less than 6 weeks to 6 months) [6–9]. In the current study, the number of days until relapse ranged between 14 and 2128 days. Because the TCRD includes an unequivocal code for never being disease-free (code: 70, Fig. 1), patients who relapsed very early (e.g., < 6 weeks) were not excluded. The interval between surgery and adjuvant radiotherapy was approximately 6 weeks. In presence of biologically aggressive tumor features (e.g., ENE), relapses may occur as early as 2 − 6 weeks after surgery. In a previous study from our group, a second FDG-PET scan before adjuvant treatment was clinically useful for early detection of residual/relapsing tumors [31]. Another investigation did not exclude patients with very early relapsing disease – which was defined as any tumor recurrence occurring between surgical resection and the beginning of scheduled post-operative RT [32]. While the adverse prognostic significance of early recurrence is in line with the current literature, the optimal cutoff for the relapse interval was not consistent across studies [6–9]; these discrepancies may be related to different study designs, patient characteristics, and statistical methodology.

On analyzing the prognostic impact of different RFs, we found that ENE – which has a decisive influence on survival outcomes in OCSCC – did not retain its independent predictive value in patients who relapsed. This surprising finding may be related to the collinearity between the presence of ENE and pN3 disease. Additionally, the prognostic impact of margin status in our patients with relapsing disease was limited. While tumor margins are a significant prognostic driver for local control and distant metastases, their impact on neck control is limited [1]. In general, we found that patients who relapsed early had a higher burden of clinicopathological RFs compared with the late relapse group. In this scenario, a thorough follow-up schedule aimed at an early detection of relapsing disease should be recommended for all patients who harbor baseline RFs.

The site of tumor relapse was found to be a key prognostic determinant; specifically, the presence of relapsing disease at distant sites was associated with dismal outcomes (5-year OS rate in the current study: 4%; Supplementary Fig. 1). The question as to whether an early detection of distant metastases may improve the outcomes of patients with OCSCC remains unanswered. We have previously shown that a second FDG-PET scan before adjuvant treatment may detect distant relapses in up to 10% of patients with OCSCC and ENE [31]. In addition, patients with known locoregional relapses should be strictly monitored for the potential occurrence of relapsing disease at distant sites [16]. At 3, 6, 9, and 12 post-treatment months, high-risk patients should undergo CT/MRI and/or FDG-PET imaging of the head and neck, thorax, and abdomen with the goal of detecting recurrent lesions [16]. Neck ultrasound, fine needle cytology, or core needle pathology (when necessary) in combination with CT/MRI/FDG PET imaging can also be helpful for detecting recurrent disease.

## Limitations

There are limitations to this study. First, the TCRD does not include regular follow-up data. While clinical and laboratory variables are prospectively collected, substantial variation in health care quality across different hospitals can exist. This shortcoming may explain why the relapse rate in our study was lower than that reported in the published literature. Second, we had no information on the prognostic impact of salvage therapy, which is an important prognostic determinant. Further research is necessary to investigate the impact of salvage therapy on survival outcomes following the identification of a relapse. Third, we had no data on the chemotherapy regimens used in the study patients. The use of different chemotherapy schemes may therefore represent a source of residual confounding that was not taken into account. Fourth, certain pathological RFs (e.g., perineural invasion, lymphatic invasion, and vascular invasion) were included in our nationwide database as of 2018 only; for that reason, their prognostic impact deserves further scrutiny. Fifth, registry-based studies are prone to unavoidable confounding or residual confounding on unmeasured variables. Sixth, we were unable to analyze the potential confounding impact of the human papillomavirus infection (HPV) status. While the role of HPV infections in the pathogenesis of OCSCC has yet to be fully elucidated, we have previously shown that HPV 16/18 E7 viral loads can predict the risk of distant metastases in patients OCSCC patients [33–35]. Further research is needed to evaluate the potential role of HPV infections in relation to the timing of tumor relapse. Finally, since the optimal cutoff used to determine early *versus* late recurrence for use in multivariable analysis was selected in univariate analysis of the same data set, we acknowledge a potential issue of statistical overfitting. Nonetheless, on analyzing the variables associated with DSS and OS in patients with early *versus* late relapses, we included the most relevant factors, and we were then able to apply both multivariable analysis and PS matching. More studies are necessary to confirm our findings and devise carefully designed surveillance strategies.

## Conclusion

Despite these limitations, these data represent a promising step in understanding the prognostic impact of the relapse interval in patients with OCSCC. After adjustment for potential confounders and PS matching, early tumor relapse was identified as an adverse predictor of survival outcomes in patients with OCSCC. Our findings are chiefly confirmatory of previous data showing that early relapse has adverse prognostic implications. Notably, of the five published studies focusing on clinical outcomes in patients with relapsing OCSCC (Table 4), two suggested that the optimal cutoff was 18 months rather than one year. While the adverse prognostic impact of early relapse was expected, we took advantage of a nationwide cohort to identify an optimal cutoff value for the relapse interval. Our data may pave the way to the prospective validation of this cutoff to guide clinical decision-making.

## Electronic supplementary material

Below is the link to the electronic supplementary material.

Supplementary Material 1

Supplementary Material 2

Supplementary Material 3: Kaplan-Meier plots of 5-year disease-specific survival (A) and overall survival (B) in patients who did not develop distant metastases *versus* those who had evidence of distant failure in the original cohort (n = 2327); Kaplan-Meier plots of 5-year disease-specific survival (C) and overall survival (D) in patients who did not develop distant metastases *versus* those who had evidence of distant failure in the propensity score-matched cohort (n = 1308)

## Data Availability

The National Health Insurance Research Dataset (NHIRD) – which is open for consultation to Taiwanese researchers – can be accessed through the Health and Welfare Data Science Center (HWDC) of the Ministry of Health and Welfare (MOHW) (http://dep.mohw.gov.tw/DOS/). The HWDC provides on-site data annotation tools and analyses services to ensure that the reported information is reliable, trustworthy, and secure. Due to legal restrictions enforced by the Taiwan government in relation to the “Personal Information Protection Act”, raw data cannot be made publicly available. At the time of writing, interested researchers can get access to the nationwide data sets used for this study by submitting a formal application to the HWDC, Department of Statistics, MOHW. More details can be found at the following URL (currently in Chinese only): http://dep.mohw.gov.tw/DOS/np-2497-113.html. Reasonable requests for obtaining data on which this study was based should be directed to Yu-Wen Wen (Telephone: +886-3211-8800 [ext. 3759], E-mail: ywwen@mail.cgu.edu.tw) – subject to a pre-existing formal permission obtained from the HWDC, MOHW.

## Abbreviations

OCSCC: oral cavity squamous cell carcinoma
NCCN: National Comprehensive Cancer Network
ENE: extra-nodal extension
CCRT: concurrent chemoradiotherapy
ND: neck dissection
RFs: risk factors
TCRD: Taiwanese Cancer Registry Database
DOI: depth of invasion
TNHIRD: Taiwanese National Health Insurance Research Dataset
MDTC: multidisciplinary team care
AJCC: American Joint Committee on Cancer
DSS: disease-specific survival
OS: overall survival
DFS: disease-free survival
STORE: Standards for Oncology Registry Entry
T: local
N: neck
M: distant
PS: Propensity score
HRs: hazard ratios
CIs: confidence intervals
RT: radiotherapy
IMRT: intensity modulated radiation therapy
VMAT: Volumetric-modulated arc therapy
AHR: average hazard ratio
SHR: subdistribution hazard ratio.
