# Supplementary material for: Early relapse is an adverse prognostic factor for survival outcomes in patients with oral cavity squamous cell carcinoma: results from a nationwide registry study
Source: BMC Cancer. 2023 Feb 7;23:126. doi: 10.1186/s12885-023-10602-1 (PMC9906940; doi:10.1186/s12885-023-10602-1)
Supplement: Supplementary file 2 — Supplementary Material 2 [file 12885_2023_10602_MOESM2_ESM.doc]

**Supplementary Table 2** Univariable and multivariable analyses of risk factors for 5-year disease-specific and overall survival in the entire study cohort (n = 2327) according to hazard ratio (HR, standard Cox regression), subdistribution hazard ratio (SHR, competing risk), and average hazard ratio (AHR, weighted Cox regression) using the day of relapse as the index date

| Risk factor | Disease-specific survival | | | | | | | | | |  | Overall survival | | | | | | | |
| --- | --- | --- | --- | --- | --- | --- | --- | --- | --- | --- | --- | --- | --- | --- | --- | --- | --- | --- | --- |
|  | Univariable  analysis | | Stepwise^a^  multivariable analysis | | Competing risk  analysis | | Univariable  analysis | | Stepwise^a^ multivariable  analysis | |  | Univariable  analysis | | Stepwise^a^  multivariable analysis | | Univariable  analysis | | Stepwise^a^ multivariable  analysis | |
|  | HR (95% CI) | *p* | HR (95% CI) | *p* | SHR (95% CI) | *p* | AHR (95% CI) | *p* | AHR (95% CI) | *p* |  | HR (95% CI) | *p* | HR (95% CI) | *p* | AHR (95% CI) | *p* | AHR (95% CI) | *p* |
| Relapse interval |  |  |  |  |  |  |  |  |  |  |  |  |  |  |  |  |  |  |  |
| Late (>330 days) | 1 |  | 1 |  | 1 |  | 1 |  | 1 |  |  | 1 |  | 1 |  | 1 |  | 1 |  |
| Early (≤330 days) | 2.27 (2.01-2.56) | <0.0001 | 1.73 (1.53-1.96) | <0.0001 | 1.73 (1.54-1.95) | <0.0001 | 2.50 (2.21-2.82) | <0.0001 | 1.81 (1.59-2.05) | <0.0001 |  | 2.22 (1.99-2.48) | <0.0001 | 1.70 (1.51-1.90) | <0.0001 | 2.50 (2.23-2.80) | <0.0001 | 1.78 (1.57-2.01) | <0.0001 |
| Tumor subsite |  |  |  |  |  |  |  |  |  |  |  |  |  |  |  |  |  |  |  |
| Lip | 1 |  | 1 |  | / |  | 1 |  | 1 |  |  | 1 |  | 1 | - | 1 |  | 1 |  |
| Tongue | 1.39 (1.01-1.91) | 0.0442 | - | ns | / | / | 1.37 (0.97-1.94) | 0.0726 | - | ns |  | 1.13 (0.86-1.50) | 0.3836 | - | ns | 1.14 (0.86-1.51) | 0.3720 | - | ns |
| Gum | 1.51 (1.08-2.12) | 0.0166 | - | ns | / | / | 1.44 (1.01-2.05) | 0.0451 | - | ns |  | 1.28 (0.95-1.73) | 0.1088 | - | ns | 1.26 (0.93-1.71) | 0.1315 | - | ns |
| Mouth floor | 1.39 (0.92-2.10) | 0.1149 | - | ns | / | / | 1.32 (0.86-2.02) | 0.2016 | - | ns |  | 1.27 (0.88-1.84) | 0.1978 | - | ns | 1.25 (0.86-1.82) | 0.2483 | - | ns |
| Hard palate | 1.54 (0.94-2.53) | 0.0847 | - | ns | / | / | 1.38 (0.85-2.25) | 0.1901 | - | ns |  | 1.57 (1.01-2.44) | 0.0455 | - | ns | 1.36 (0.91-2.04) | 0.1324 | - | ns |
| Buccal | 1.21 (0.88-1.67) | 0.2421 | - | ns | / | / | 1.17 (0.84-1.65) | 0.3537 | - | ns |  | 1.01 (0.76-1.34) | 0.9485 | - | ns | 0.997 (0.75-1.33) | 0.9584 | - | ns |
| Retromolar | 1.26 (0.86-1.85) | 0.2291 | - | ns | / | / | 1.23 (0.82-1.84) | 0.3082 | - | ns |  | 1.10 (0.78-1.54) | 0.5940 | - | ns | 1.12 (0.79-1.59) | 0.5122 | - | ns |
| Other sites | 1.02 (0.65-1.59) | 0.9404 | - | ns | / | / | 1.09 (0.67-1.78) | 0.7272 | - | ns |  | 1.09 (0.74-1.61) | 0.6614 | - | ns | 1.20 (0.79-1.83) | 0.3957 | - | ns |
| Sex |  |  |  |  |  |  |  |  |  |  |  |  |  |  |  |  |  |  |  |
| Men | 0.94 (0.81-1.17) | 0.7630 | - | ns | / | / | 0.96 (0.79-1.18) | 0.7050 | - | ns |  | 1.00 (0.84-1.19) | 1.0000 | - | ns | 0.97 (0.80-1.17) | 0.7824 | - | ns |
| Women | 1 |  | 1 |  | / |  | 1 |  | 1 |  |  | 1 |  | 1 |  | 1 |  | 1 |  |
| Age (years) |  |  |  |  |  |  |  |  |  |  |  |  |  |  |  |  |  |  |  |
| <65 | 1 |  | 1 |  | 1 |  | 1 |  | 1 |  |  | 1 |  | 1 |  | 1 |  | 1 |  |
| ≥65 | 1.15 (0.99-1.33) | 0.0604 | 1.23 (1.07-1.43) | 0.0048 | 1.23 (1.05-1.45) | 0.0117 | 1.22 (1.04-1.43) | 0.0147 | 1.33 (1.12-1.58) | 0.0009 |  | 1.20 (1.05-1.38) | 0.0068 | 1.31 (1.14-1.50) | 0.0001 | 1.27 (1.10-1.47) | 0.0011 | 1.41 (1.20-1.65) | <0.0001 |
| Pathologic T status |  |  |  |  |  |  |  |  |  |  |  |  |  |  |  |  |  |  |  |
| T1 | 1 |  | 1 | - | / |  | 1 |  | 1 |  |  | 1 |  | 1 |  | 1 |  | 1 |  |
| T2 | 1.77 (1.44-2.17) | <0.0001 | - | ns | / | / | 1.90 (1.54-2.35) | <0.0001 | - | ns |  | 1.78 (1.48-2.15) | <0.0001 | - | ns | 1.97 (1.63-2.39) | <0.0001 | - | ns |
| T3 | 2.52 (2.03-3.11) | <0.0001 | - | ns | / | / | 2.78 (2.24-3.44) | <0.0001 | - | ns |  | 2.59 (2.13-3.15) | <0.0001 | - | ns | 2.93 (2.40-3.57) | <0.0001 | - | ns |
| T4 | 3.41 (2.81-4.13) | <0.0001 | - | ns | / | / | 4.01 (3.29-4.90) | <0.0001 | - | ns |  | 3.84 (3.21-4.58) | <0.0001 | - | ns | 4.53 (3.79-5.42) | <0.0001 | - | ns |
| Pathologic N status |  |  |  |  |  |  |  |  |  |  |  |  |  |  |  |  |  |  |  |
| pN0 | 1 |  | 1 |  | 1 |  | 1 |  | 1 |  |  | 1 |  | 1 |  | 1 |  | 1 |  |
| pN1 | 1.85 (1.57-2.19) | <0.0001 | 1.21 (1.001-1.46) | 0.0483 | 1.22 (0.99-1.51) | 0.0665 | 1.96 (1.64-2.33) | <0.0001 | 1.23 (0.99-1.53) | 0.0655 |  | 1.79 (1.53-2.09) | <0.0001 | 1.26 (1.06-1.50) | 0.0090 | 1.94 (1.64-2.30) | <0.0001 | 1.30 (1.05-1.61) | 0.0159 |
| pN2 | 2.31 (2.01-2.65) | <0.0001 | 1.42 (1.20-1.69) | <0.0001 | 1.42 (1.18-1.72) | 0.0003 | 2.64 (2.20-3.18) | <0.0001 | 1.50 (1.21-1.86) | 0.0002 |  | 2.30 (2.01-2.62) | <0.0001 | 1.34 (1.14-1.58) | 0.0005 | 2.56 (2.22-2.95) | <0.0001 | 1.37 (1.13-1.66) | 0.0012 |
| pN3 | 3.19 (2.83-3.61) | <0.0001 | 1.67 (1.42-1.96) | <0.0001 | 1.66 (1.39-1.99) | <0.0001 | 3.56 (3.11-4.07) | <0.0001 | 1.69 (1.40-2.04) | <0.0001 |  | 3.44 (3.07-3.86) | <0.0001 | 1.70 (1.46-1.98) | <0.0001 | 3.86 (3.40-4.38) | <0.0001 | 1.72 (1.44-2.06) | <0.0001 |
| Pathologic stage |  |  |  |  |  |  |  |  |  |  |  |  |  |  |  |  |  |  |  |
| I | 1 |  | 1 |  | 1 |  | 1 |  | 1 |  |  | 1 |  | 1 |  | 1 |  | 1 |  |
| II | 1.42 (1.09-1.84) | 0.0090 | 1.22 (0.93-1.59) | 0.1446 | 1.22 (0.95-1.57) | 0.1149 | 1.45 (1.12-1.89) | 0.0053 | 1.24 (0.94-1.62) | 0.1238 |  | 1.46 (1.16-1.85) | 0.0015 | 1.29 (1.02-1.64) | 0.0335 | 1.55 (1.23-1.95) | 0.0002 | 1.35 (1.05-1.72) | 0.0184 |
| III | 2.37 (1.84-3.05) | <0.0001 | 1.60 (1.20-2.13) | 0.0015 | 1.58 (1.20-2.10) | 0.0013 | 2.57(2.01-3.30) | <0.0001 | 1.70 (1.26-2.29) | 0.0005 |  | 2.26 (1.79-2.84) | <0.0001 | 1.58 (1.22-2.05) | 0.0006 | 2.53 (2.02-3.17) | <0.0001 | 1.70 (1.29-2.23) | 0.0002 |
| IV | 4.17 (3.33-5.22) | <0.0001 | 1.69 (1.26-2.27) | 0.0005 | 1.68 (1.26-2.25) | 0.0004 | 5.13 (4.11-6.42) | <0.0001 | 1.94 (1.42-2.64) | <0.0001 |  | 4.44 (3.63-5.43) | <0.0001 | 1.99 (1.54-2.55) | <0.0001 | 5.58 (4.59-6.79) | <0.0001 | 2.23 (1.70-2.92) | <0.0001 |
| Tumor differentiation |  |  |  |  |  |  |  |  |  |  |  |  |  |  |  |  |  |  |  |
| Well | 1 |  | 1 | - | / |  | 1 |  | 1 |  |  | 1 |  | 1 |  | 1 |  | 1 |  |
| Moderately | 1.26 (1.10-1.44) | 0.0009 | - | ns | / | / | 1.34 (1.15-1.56) | 0.0001 | - | ns |  | 1.25 (1.10-1.42) | 0.0004 | 1.02 (0.89-1.15) | 0.8193 | 1.32 (1.16-1.50) | <0.0001 | 1.03 (0.89-1.19) | 0.7128 |
| Poorly | 1.78 (1.50-2.11) | <0.0001 | - | ns | / | / | 1.94 (1.62-2.34) | <0.0001 | - | ns |  | 1.86 (1.59-2.18) | <0.0001 | 1.28 (1.09-1.51) | 0.0032 | 2.04 (2.41-8.27) | <0.0001 | 1.29 (1.08-1.54) | 0.0048 |
| Undifferentiated | 5.45 (0.77-38.85) | 0.0906 | - | ns | / | / | 11.61 (2.48-54.44) | 0.0019 | - | ns |  | 4.95 (0.69-35.29) | 0.1106 | 1.29 (0.18-9.23) | 0.7985 | 10.47 (2.20-49.87) | 0.0032 | 2.50 (0.52-11.97) | 0.2517 |
| Depth of invasion |  |  |  |  |  |  |  |  |  |  |  |  |  |  |  |  |  |  |  |
| <10 mm | 1 |  | 1 |  | 1 |  | 1 |  | 1 |  |  | 1 |  | 1 |  | 1 |  | 1 |  |
| ≥10 mm | 2.01 (1.80-2.24) | <0.0001 | 1.17 (1.01-1.35) | 0.0415 | 1.14 (1.00-1.31) | 0.0503 | 2.16 (1.91-2.46) | <0.0001 | 1.16 (0.99-1.34) | 0.0593 |  | 2.13 (1.92-2.35) | <0.0001 | 1.23 (1.09-1.38) | 0.0007 | 2.27 (2.04-2.53) | <0.0001 | 1.22 (1.07-1.40) | 0.0039 |
| Margin status |  |  |  |  |  |  |  |  |  |  |  |  |  |  |  |  |  |  |  |
| <5 mm | 1.09 (0.98-1.20) | 0.1079 | - | ns | / | / | 1.14 (1.01-1.28) | 0.0275 | - | ns |  | 1.09 (0.99-1.19) | 0.0810 | - | ns | 1.12 (1.01-1.24) | 0.0313 | - | ns |
| ≥5 mm | 1 |  | 1 | - | / |  | 1 |  | 1 |  |  | 1 |  | 1 | - | 1 |  | 1 |  |
| Extra-nodal extension |  |  |  |  |  |  |  |  |  |  |  |  |  |  |  |  |  |  |  |
| No | 1 |  | 1 | - | / |  | 1 |  | 1 |  |  | 1 |  | 1 | - | 1 |  | 1 |  |
| Yes | 2.27 (2.04-2.51) | <0.0001 | - | ns | / | / | 2.38 (2.10-2.69) | <0.0001 | - | ns |  | 2.44 (2.21-2.69) | <0.0001 | - | ns | 2.58 (2.31-2.88) | <0.0001 | - | ns |
| Treatment modality |  |  |  |  |  |  |  |  |  |  |  |  |  |  |  |  |  |  |  |
| S alone | 1 |  | 1 |  | 1 |  | 1 |  | 1 |  |  | 1 |  | 1 | - | 1 |  | 1 |  |
| S plus CT | 2.33 (2.08-2.61) | <0.0001 | 1.17 (1.01-1.35) | 0.0415 | 1.17 (1.003-1.36) | 0.0461 | 2.51 (2.22-2.85) | <0.0001 | 1.12 (0.95-1.33) | 0.1846 |  | 2.36 (2.13-2.62) | <.0001 | - | ns | 2.58 (2.31-2.87) | <0.0001 | - | ns |
| + S plus RT |  |  |  |  |  |  |  |  |  |  |  |  |  |  |  |  |  |  |  |
| + S plus CT and RT |  |  |  |  |  |  |  |  |  |  |  |  |  |  |  |  |  |  |  |
| Distant relapse |  |  |  |  |  |  |  |  |  |  |  |  |  |  |  |  |  |  |  |
| No | 1 |  | 1 |  | 1 |  | 1 |  | 1 |  |  | 1 |  | 1 |  | 1 |  | 1 |  |
| Yes | 3.15 (2.83-3.49) | <0.0001 | 2.04 (1.82-2.29) | <0.0001 | 2.05 (1.79-2.34) | <0.0001 | 3.58 (3.17-4.05) | <0.0001 | 2.28 (1.98-2.62) | <0.0001 |  | 3.82 (3.45-4.23) | <0.0001 | 2.55 (2.29-2.85) | <0.0001 | 4.36 (3.88-4.91) | <0.0001 | 2.83 (2.49-3.22) | <0.0001 |

*HR* hazard ratio, *SHR* subdistribution hazard ratio, *AHR* average hazard ratio, *CI* confidence interval, *ns* not significant, *S* surgery, *CT* chemotherapy, *RT* radiotherapy

^a^ In univariable analyses, each covariate was added separately to the Cox regression model. In multivariable analysis, all covariates were entered into the Cox regression using a stepwise procedure for variable selection. The symbol “-” indicates that the corresponding variable was excluded from the Cox regression model after applying the stepwise selection procedure.
