# Supplementary figures and images for: Early relapse is an adverse prognostic factor for survival outcomes in patients with oral cavity squamous cell carcinoma: results from a nationwide registry study
Source: BMC Cancer. 2023 Feb 7;23:126. doi: 10.1186/s12885-023-10602-1 (PMC9906940; doi:10.1186/s12885-023-10602-1)

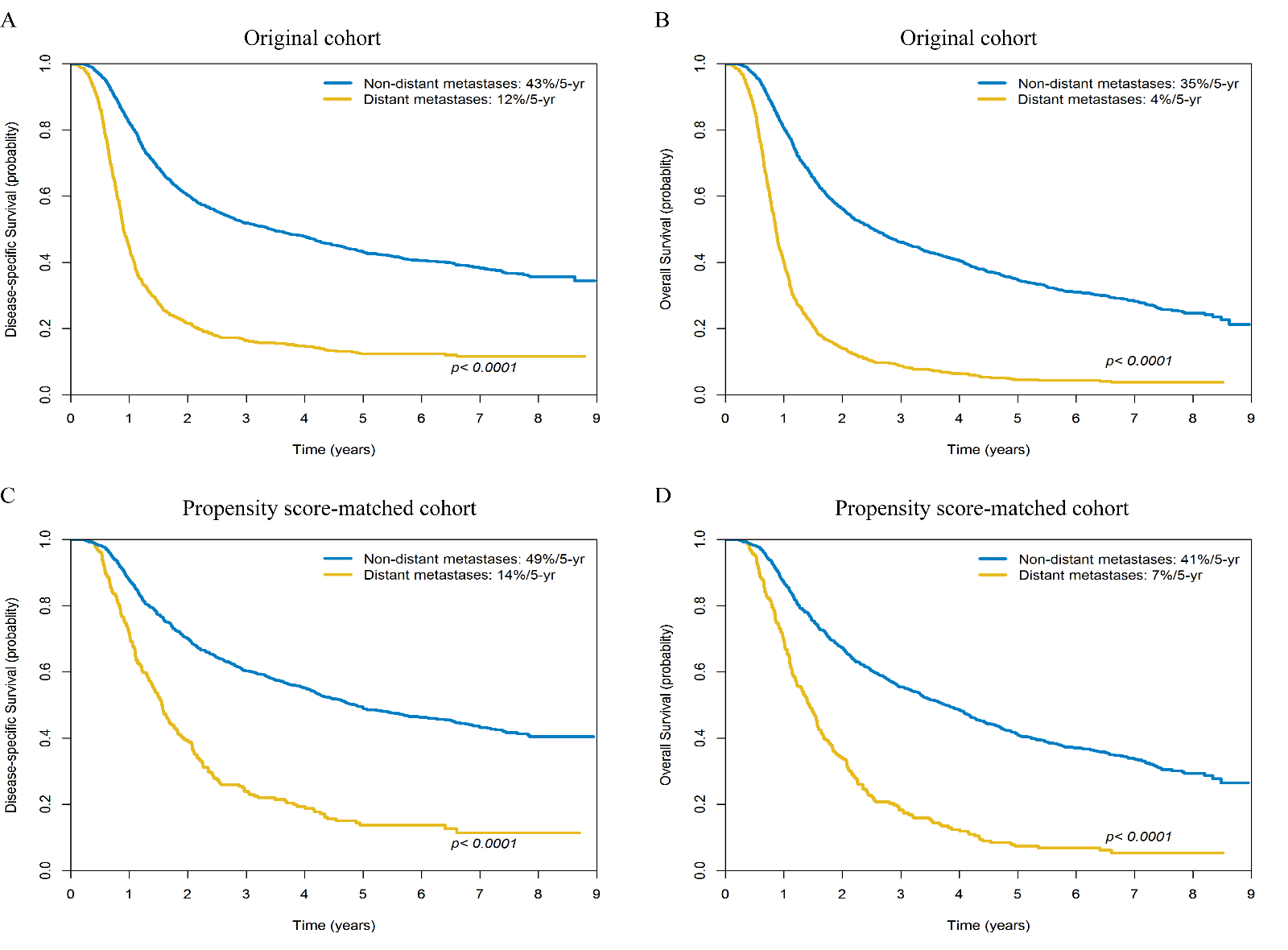

Supplement: Supplementary file 3 — Supplementary Material 3: Kaplan-Meier plots of 5-year disease-specific survival (A) and overall survival (B) in patients who did not develop distant metastases versus those who had evidence of distant failure in the original cohort (n = 2327); Kaplan-Meier plots of 5-year disease-specific survival (C) and overall survival (D) in patients who did not develop distant metastases versus those who had evidence of distant failure in the propensity score-matched cohort (n = 1308) [file 12885_2023_10602_MOESM3_ESM.png]
